# Intra- and Intermuscular Coherence and Body Acceleration Control in Older Adults during Bipedal Stance

**DOI:** 10.3390/geriatrics6040114

**Published:** 2021-12-10

**Authors:** Tadayoshi Minamisawa, Noboru Chiba, Eizaburo Suzuki

**Affiliations:** 1 Department of Physical Therapy, Yamagata Prefectural University of Health Sciences, 260 Kamiyanagi, Yamagata 990-2212, Japan; esuzuki@yachts.ac.jp; 2 Department of Occupational Therapy, Yamagata Prefectural University of Health Sciences, 260 Kamiyanagi, Yamagata 990-2212, Japan; nchiba@yachts.ac.jp

**Keywords:** bipedal stance, EMG coherence, COM acceleration, aging

## Abstract

Our aim was to clarify the effect of aging on the coherence of electromyograms of plantar flexor pairs during bipedal stance and to clarify the relationship between coherence and center-of-mass acceleration (COMacc). The subjects were 16 adults and 18 older adults. Intra- and intermuscular coherence and phase analyses were used to analyze the muscle pairs of bilateral and unilateral plantar flexor muscle groups. The relationship between coherence value and anterior–posterior COMacc of the plantar flexor muscle pairs was also examined to determine whether the connectivity of the lower limb muscle pairs is functionally important. The older adults showed higher coherence in the frequency range of 0–4 Hz for muscle pairs than the younger adults. In phase analysis, the older adults showed a phase difference between bilateral heteronymous muscle pairs in the frequency range of 0–6 Hz, which was one of the characteristics not seen in the younger adults. Correlation analysis showed that all the muscle pairs were moderately correlated with COMacc in the older adults. Not only does aging affects the organization of the bilateral and unilateral postural muscle activity of the plantar flexors during bipedal stance, but such organization may also be related to the increased COMacc characteristics of older adults.

## 1. Introduction

The problem of balance loss due to weak physical function is one of the main health problems faced by older adults. Maintaining balance control in bipedal stance is essential for the performance of activities of daily living; therefore, many evaluation techniques have been proposed to objectify postural control [1,2]. In particular, it is very common and important to examine muscle activity while standing because the central nervous system (CNS) generates motor commands based on the integrated sensory cues of body fluctuations, following which muscle activities occur to maintain an upright posture [3]. Furthermore, even for a seemingly simple task such as upright posture, limb interaction is essential to maintain postural stability, and humans have neural circuits to maintain limb coordination [4]. Previous studies on lower limb connectivity during standing have shown that there is a significant EMG–EMG coherence difference between the plantar flexors of both lower limbs in the frequency range of the muscle network in younger and older adults [5,6]. The ability to couple both lower limbs in bipedal stance can be achieved at the spinal cord level, most likely via sensory feedback from peripheral mechanoreceptors in each lower limb [7]. In addition, proprioception, the sense of limb and body position, is important for the timing of rhythmic movements such as walking, as well as coordination of muscle activity across joints [8], and these senses are largely relayed and processed in the dorsal spinal cord. Furthermore, against the background of such mechanisms, several studies have discussed the relationship between contralateral homologous muscle and unilateral heteronymous muscle in lower limb muscle modulation during bipedal stance [5,6], but the relationship between the contralateral heteronymous muscles has not yet been clarified. Multisensory integration of visual, vestibular, somatosensory, and auditory inputs is used to control standing balance, and coordination of postural muscles throughout the body is used. Furthermore, among the postural muscles, the soleus (SOL) and medial gastrocnemius (MG) muscles, in particular, play an important role as the primary antigravity muscles for maintaining an upright posture. It is interesting to see how this heteronymous pair of muscles relate to each other across the lower limbs, because differences in EMG responses (i.e., intermittent or sustained activity) have also been observed between MG and SOL in functional tasks such as weight shifting and walking [9]. Co-contraction of co-activation muscles, and not only homonymous muscle pairs but also heteronymous muscles across both lower limbs, may interfere with joint motion redundancy of the lower limbs in the standing posture, where joint motion freedom is high [10]. Whether these interlimb muscle networks are related in standing has not been systematically examined. Bilateral coupling of the limbs necessary for efficient and stable work and activities of daily living may be more important for older adults. In other words, understanding the interlimb connectivity may provide an opportunity to optimize physical function recovery and quality of life in older adults in the future. The first purpose of this study was to investigate the age dependence of intermuscular coherence of both lower limbs in bipedal stance as a function of postural stability.

Moreover, it is also important to know whether such a muscle network is functionally important. It should be noted that co-contraction strategies in older adults can stiffen both joints [10] and cause loss of balance. Therefore, the second purpose of this study was to investigate whether intra- and intermuscular coherence (IMC) is related to anterior–posterior center-of-mass acceleration (COMacc) [11,12,13], which is often used as a balance index. If this is revealed, the aforementioned index may become a new paradigm and make more interesting contributions in fall prevention and understanding standing control. Our hypothesis for intermuscular coherence, referring to a previous study [14], was that age-related co-activation of contralateral heteronymous muscle pairs in both lower limbs would result in increased coherence values. Furthermore, since motor coordination of both lower limbs is necessary to maintain a standing posture, we hypothesized that EMG coherence values in the elderly might be more strongly related to COMacc [2].

## 2. Materials and Methods

### 2.1. Subjects

This study enrolled 16 healthy younger adults (age: 20.4 ± 0.6 years; height: 165.9 ± 8.3 cm; weight: 58.3 ± 6.3 kg) and 18 healthy older adults (age: 72.9 ± 3.6 years; height: 162.0 ± 8.2 cm; weight: 62.9 ± 8.6 kg). Individuals with a history of musculoskeletal injuries or diseases and those with neurological disorders were excluded. Prior to participation, the subjects received a written description of the research protocol and provided informed consent. 

### 2.2. Measurement

The participants were asked to stand barefoot on two force plates (Type-9287A; Kistler, 60 × 90 cm; Winterthur, Switzerland), with each foot on a separate force plate, with a comfortable arm and stance width, and to keep their eyes open. We also carefully observed the participants to make sure that the distance between their chosen foot widths did not exceed their shoulder width. The feet position was marked with tape to ensure that the same position was used during all trials. To record muscle activity, wireless surface EMG sensors (Trigno Wireless EMG System, Delsys, Boston, MA, USA) were attached bilaterally to the medial gastrocnemius and soleus muscles. The duration of each trial was approximately 60 s, with a 1-min interval resting time between the trials [2]. All data were collected at a sampling frequency of 1000 Hz, and the signals were input into a personal computer for analysis. For this analysis, the center of pressure in the anterior–posterior direction (COPap) and the center of pressure in the medial–lateral direction (COPml) of each lower limb were calculated from the two force plates with the aim of clarifying the asymmetry of the kinematic characteristics of both lower limbs.

### 2.3. Data Processing

To estimate intermuscular coherence, EMG signals were high-pass filtered at a frequency of 20 Hz using a fourth-order Butterworth filter with zero phase-lag and rectified using Hilbert transform [5]. Note that since frequencies below 10 Hz have been reported as one of the characteristic frequency bands of intermuscular coherence, we focused our analysis on this frequency band in this study. All the COP data, the direct current (DC) offset (mean amplitude displacement from zero), which were obtained from the force plate, were removed, and the data were low-pass filtered using a fourth-order, zero-phase-lag Butterworth filter with a cut-off frequency of 20 Hz [15]. All the data analyses were performed using the NI DIAdem 2020 (National Instrument, Austin, TX, USA).

### 2.4. Coherence Analysis

The extent to which modulations in both lower limb muscle activities were correlated was analyzed in the frequency domains. Spectral coherence, a common oscillation between the two EMG signals, was identified in the frequency range of 0–10 Hz. EMG–EMG coherence among (1) bilateral SOL (right and left SOLs: SOLr–SOLl), (2) bilateral MG (right and left MGs: MGr–MGl), (3) unilateral (right) SOL and MG (SOLr–MGr), (4) unilateral (left) SOL and MG (SOLl–MGl), (5) contralateral heteronymous muscle (MGl–SOLr), and (6) contralateral heteronymous muscle (MGr–SOLl) muscles was calculated. Note that we defined (1) and (2) as the contralateral homologous muscle group (Group 1), (3) and (4) as the unilateral heteronymous muscle group (Group 2), and (5) and (6) as the contralateral heteronymous muscle group (Group 3). Coherence between two signals is a normalized measure of the linear correlation between signals in the frequency domain; the values range from 0 to 1, where 1 indicates perfect linear association and 0 indicates complete absence of linear association. In this study, the phase values are limited to −180° to +180°: A positive phase indicates that the target signal has advanced and led to the reference signal. Power spectral density and intermuscular coherence of the EMG envelopes was estimated between pairs of concatenated EMG data using Welch’s method with a window length of 2500 points and an overlap of 50%.

$$\left|C_{xy}\left(f\right)\right|=\frac{{\left|S_{xy}\left(f\right)\right|}^{2}}{S_{xx}\left(f\right)S_{yy}\left(f\right)}$$

where *R_xy_*(*f*) is the CPSD, and *S_xx_*(*f*) and *S_yy_*(*f*) represent the PSD of both input signals *x*(*t*) and *y*(*t*), i.e., any pairwise combination of the investigated muscles, respectively [16]. The within- and between-limbs coherence was analyzed from 0 to 10 Hz, and the average value of each 2 Hz band was calculated; these values were then compared between the younger and older adults. Moreover, coherence is considered to be significant if the resulting value lies above the confidence level (CL) [17].

$$CL\left(\alpha \right)=1-{\left(1-\alpha \right)}^{\frac{1}{n-1}}$$

where *n* is the number of segments, and α is the desired level of confidence. We considered coherence to be significant above the 95% confidence limit. In this study, the threshold coherence value was set to 0.063.

### 2.5. Cross-Correlation Function Analysis for COP

In the time-domain analysis, the center of pressure (COP) in the anterior–posterior direction (COPap) and the center of pressure in the medial–lateral direction (COPml) of both legs were calculated from the two force plates, respectively, with the aim of clarifying the correlation between the trajectories of the COP of the left and right legs in time variation. Furthermore, the correlation coefficients of the COPs of both lower limbs at zero lag were determined by normalized cross-correlation function (CCF) analysis. If the signals were identical or completely different, the correlation coefficient was set to 1 or 0, respectively.

First, it was important to calculate the one-sided cross-correlation *R_xy_* for the *x* (right foot of COP) and *y* (left foot of COP) signals. *N* is the minimum number of data points for *x* and *y*. The cross-correlation function (*R_xy_*(*t*)) was defined as follows:

$$R_{xy}=\frac{1}{N}\begin{array}{c} \overset{N}{\underset{j=1}{\sum }}x_{j}+i-1\cdot y_{j} \end{array},\;1\leq i\leq W$$

where *W* is the number of samples in the windows, and j represents the samples of the windows.

Furthermore, the normalized cross-correlation function (*R_xy_,norm*) for the two signals *x* and *y* is calculated as:

$$R_{xy,norm}=\frac{R_{xy}}{\sqrt{\frac{1}{N}\overset{N}{\underset{j=1}{\sum }}x_{j}{}^{2}\cdot \frac{1}{N}\overset{N}{\underset{j=1}{\sum }}y_{j}{}^{2}}}$$

### 2.6. Correlation Analysis for the Coherence Value and COMacc

To clarify the relationship between the coherence value and the COMacc, the ground reaction force data of anterior–posterior (Fap) of the right and left lower limbs, which were collected separately, were recorded as the total Fap and divided by the weight of each participant to obtain the COMacc. The horizontal linear COMacc can be estimated using force plate recordings according to the following equation:

COMacc (*t*) = *Fap (t)⁄m*

where *t* is the sampling point, Fap is the ground reaction force recorded in the horizontal direction, and *m* is the mass of the body [18].

### 2.7. Statistical Analysis

EMG coherence data and phase analysis data were statistically analyzed using repeated measures analysis of variance to compare the effect of aging (younger and older adults). Since this study focused on the aging effect, only the results between groups will be presented. Statistical significance was set at a level of *p* < 0.05. CCF data (COPap and COPml) were calculated using the left and right lower-limb COP and were compared between the two groups (younger and older) using the unpaired *t*-test for the mean value of the correlation coefficient for the participant at zero time-lag, with a statistical significance level of *p* < 0.05. Moreover, Pearson’s correlation coefficient (r) was used to determine the coherence value for each frequency band in EMG coherence and for the correlation coefficient for COMacc (*p* < 0.05). The strength of the relationship between COMacc and coherence values is defined as low when the coefficient (r) is between 0.1 and 0.3, medium when it is between 0.3 and 0.5, and high when it is greater than 0.5. Statistical analysis was performed using OriginPro 2017 (OriginLab, Northampton, MA, USA). 

## 3. Results

Since frequencies below 10 Hz have been reported as one of the characteristic frequency bands of intermuscular coherence, we focused our analysis on this frequency band in this study.

### 3.1. Coherence Analysis

**Figure 1:**
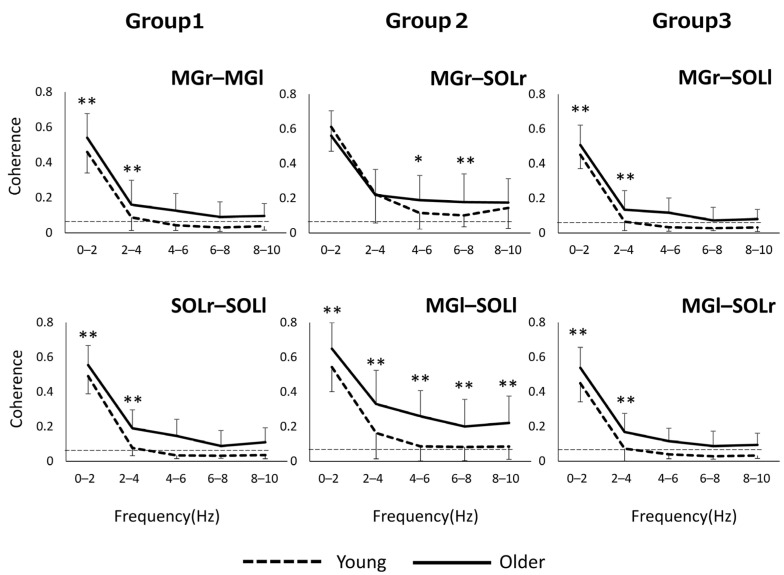
In Groups 1 and 3, the older group showed significantly higher values than the younger adults in the frequency band of 0–4 Hz (Figure 1). In Group 2, the older group had higher values than the younger one in all frequency bands within 10 Hz for the left limb muscle pair and in the 4–8 Hz frequency band for the right limb muscle pair. * *p* < 0.05, ** *p* < 0.01.

Regarding the values of inter- and intramuscular coherence, in the case of older adults, CL values within 10 Hz reached significant levels in all muscle pairs, while in the case of the younger adults, significance levels were below 4 Hz in most muscle pairs (Figure 1). 

### 3.2. Phase Analysis

**Figure 2:**
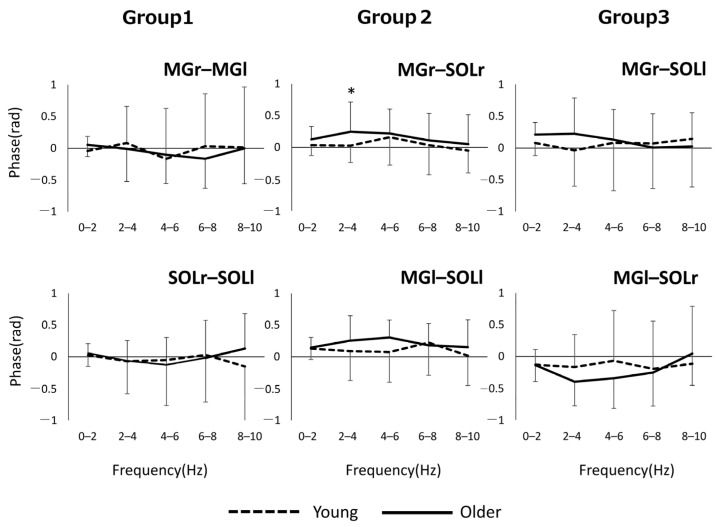
Phase plots of bilateral homonymous, unilateral heteronymous, and bilateral heteronymous muscle pairs compared between groups. * *p* < 0.05. Abbreviations: MG, medial gastrocnemius; SOL, soleus; r and l, respectively, right limb and left limb.

**Figure 3:**
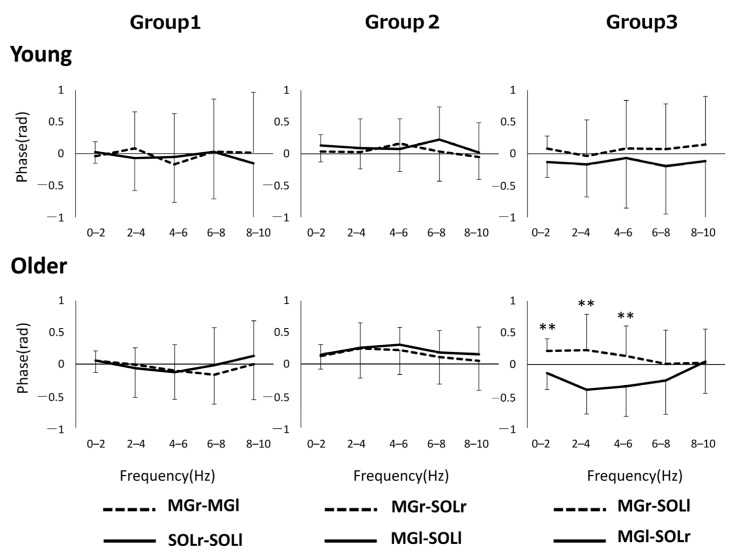
Pooled phase plots for each muscle pair of bilateral homonymous, unilateral heteronymous, and bilateral heteronymous for each group are shown. ** *p* < 0.01. Abbreviations: MG, medial gastrocnemius; SOL, soleus; r and l, respectively, right limb and left limb.

Figure 2 shows the phase–frequency plot of the muscle pairs due to the aging effect. The phase–frequency plot is almost flat around 0 rad for most of the muscle pairs in both Group 1 and Group 3, but there is a significant difference between the two groups for MGr–SOLr in Group 2 (*p* = 0.04) (Figure 2). Figure 3 shows the results of the phase difference between the muscle pairs (Group 1–Group 3) and a comparison within groups. The within-group phase–frequency plots show that both muscle pairs are close to 0 rad in the younger group (Figure 3). On the other hand, there is a phase difference between MGr–SOLl and MGl–SOLr in the older adults in the frequency ranges of 0–2 Hz (*p* = 0.001), 2–4 Hz (*p* < 0.001), and 4–6 Hz (*p <* 0.001) (Figure 3).

### 3.3. Synchronization of COP Motion between Both Feet

**Figure 4:**
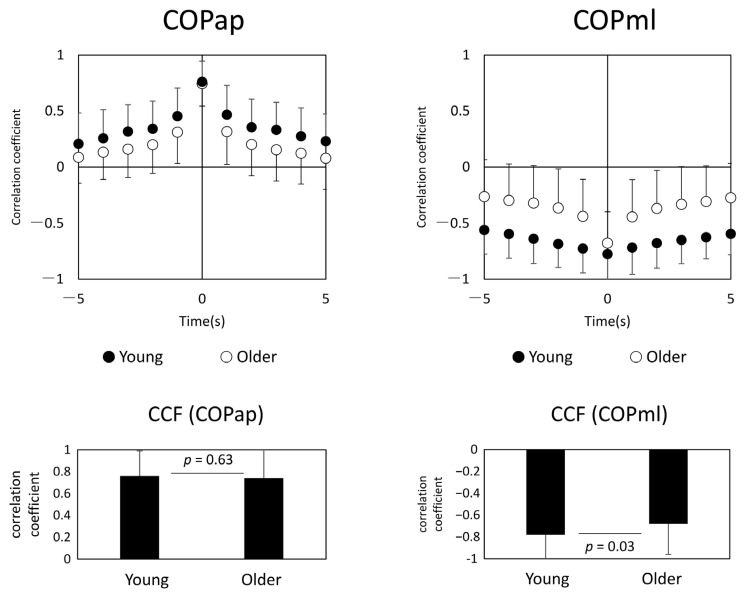
Center of pressure cross-correlation function (CCF) analysis (**left panel**: younger age group, **right panel**: older age group). The **upper panel** shows the plot of CCF, and the **lower panel** shows the results of the *t*-test between the two groups. Abbreviations: COPap, center of pressure in the anterior–posterior direction; COPml, center of pressure in the medial–lateral direction.

In general, AP COPs of both legs were positively correlated, and ML COPs were negatively correlated (Figure 4). Younger and older subjects showed the highest synchronization at zero time-lag. The correlation coefficients and standard deviations (in parentheses) between COPap and COPml for both lower limbs were r = 0.76 (0.18) for the younger and r = 0.74 (0.20) for the older group in the AP direction, and r = −0.78 (0.23) for the younger and r = −0.68 (0.28) for the older adults in the ML direction. When the correlation coefficients at zero time-lag for each group were compared by an unpaired *t*-test, there was no significant difference for COPap at *p* = 0.63; COPml showed a significant difference between both groups at *p* = 0.03.

### 3.4. Correlation Analysis for the Coherence Value and COMacc

**Figure 5:**
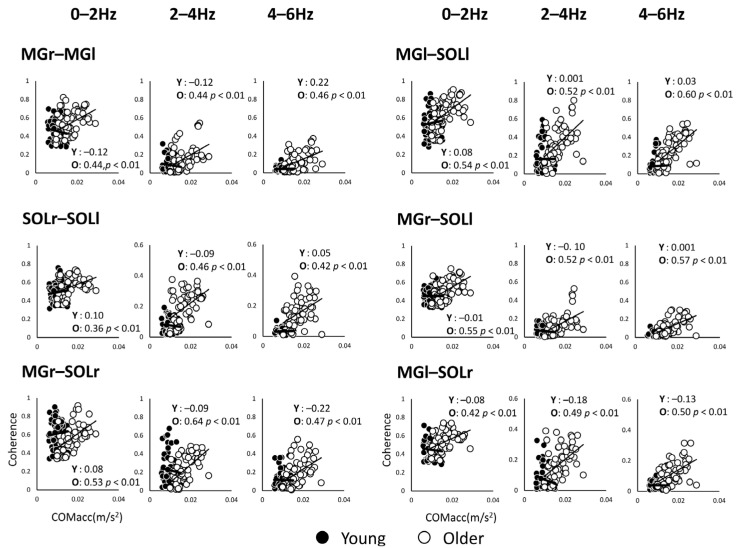
Correlation analysis between coherence values of muscle pairs and acceleration of the center of mass in the anterior–posterior direction for each of the two groups, plotted for each frequency band. The open black circles represent the younger adults (Y) and the solid circles represent the older adults (O). Abbreviations: MG, medial gastrocnemius; SOL, soleus; r and l, respectively, right limb and left limb.

Since the mean power frequency of the COMacc in the older adults is below 2 Hz [13], the correlation analysis was limited to a lower frequency band, so we proceeded to analyze within 6 Hz. The coherence values of the muscle pairs and COMacc in the anterior–posterior direction for every 2 Hz for the frequency band of 0–6 Hz are shown in Figure 5. The correlation coefficients between COMacc and intermuscular coherence in younger adults were low, while in older adults, the coherence between similar muscles in the left and right lower limbs had moderate correlation coefficients in all frequency bands compared.

## 4. Discussion

The results of this study show that the signal synchronization level within 1–10 Hz increased significantly with age, even for heterogeneous muscle groups between the two lower limbs. In addition, the results for Group 1 (contralateral homogeneous muscle groups) and Group 2 (unilateral heterogeneous muscle groups) were similar to previous studies [14,19]. While neural pathways in the human spinal cord have been shown to allow direct communication between homonymous muscles of the lower limbs [20], unfavorable synchronization [10] in older adults appears to be integrated in a wide range of muscle pairs, including pairs of heteronymous muscles [9]. As a neural mechanism for this age-related change in the level of co-contraction, a decrease in Ia afferent input into the soleus muscle [19] or a decrease in intrinsic sensation seen in older adults is also thought to be related to co-contraction [21]. 

As a characteristic of older adults, a phase difference was observed between the heteronymous muscle pairs of both lower limbs (i.e., between MGr–SOLl and MGl–SOLr) based on the phase analysis of muscle pairs, which is one of the features not seen in younger adults. Specifically, the phase was positive for MGl–SOLr and negative for MGr–SOLl, which can be interpreted as MGr preceding the muscle activity of SOLl and, similarly, SOLr preceding the muscle activity of MGl. In other words, this suggests that asynchronous activity preceded by the right lower limb muscle activities occurs in the crossed heteronymous muscle pairs between the lower limbs in older adults. In older adults, load asymmetry in stance has been noted [22], and lower limb muscle strength, loss of position sense, and co-contraction may affect the load dependence of the unilateral lower limb [23]. An important aspect of such a phase difference seen in Group 3 is that this index may be related to the significantly lower correlation coefficient (Figure 4) of COPml in the older adults. Thus, the gastrocnemius muscle, which usually plays an important role in sagittal plane control during standing, may contribute not only to sagittal plane control, but also to lateral stabilization of the body by generating foot internal rebound torque [24]. Phase differences in the muscle activity of the plantar flexors of both lower limbs may result in loading asymmetry and reduced synchronization of the COP. It is a well-known fact that the ability to control standing in the ML direction in older adults is related to the risk of falling [25,26,27], and if changes in the phase difference between muscle groups are behind this, analyzing inter-muscular coherence may be used as an assessment value to prevent future falls. On the other hand, it is still unclear why the phase difference was observed in the heteronymous muscle pairs. One possibility is that MG and SOL have different functions. In the frontal plane during standing, each lower limb alternately loads and unloads [28], generating forces in opposite phases. The SOL and MG have different functions; the SOL function continuously to counteract gravitational loads, whereas the gastrocnemius tends to work intermittently to stabilize the body [9]. Since human stance is controlled by two lower limbs, it is possible that sustained and intermittent control by the leg muscles alternates in both lower limbs during repeated loading and unloading. In any case, further studies are needed to clarify the potential causes of the underlying problem of temporal and frequency asynchrony in muscle activity. 

The relationship between COMacc and coherence showed different correlations in younger and older adults. A moderate positive correlation between intermuscular coherence and COMacc was observed in the older adults, suggesting that bilateral coordination of the lower limbs by the intermuscular coherence may be a clinically important indicator of motor control in bipedal stance. Interlimb coordination is an intrinsic property of human neural circuitry; the COM is relatively high in the bipedal stance, and from a functional perspective, interlimb coordination is necessary to maintain the COM on the supporting base plane [29,30]. In tasks characterized by a free kinetic chain, such as stance, co-contraction of lower limb muscles across both limbs, as observed in the older adults, may increase body stiffness [31] and inhibit redundancy of lower limb joint movements in the sagittal plane [10]. If there is a relationship between inter- and intramuscular coherence and COMacc, the significance of investigating coordination in bipedal stance will increase, suggesting that IMC may become a more important indicator in future aging research. Furthermore, by including the contralateral heteronymous muscles between the lower limbs in the analysis, we have the potential to evaluate the coordination of both lower limbs in the sagittal or frontal plane in the bipedal stance. Therefore, we believe that it will be possible to evaluate multiple aspects of standing in a simpler measurement setting in clinical assessments.

## 5. Limitations

Although only plantar flexors were measured in this study, it has been reported that the contribution of the hip joint is essential for the motor control of the frontal plane [28]. Therefore, it is unclear to what extent the plantar flexors contributed to the control. In addition, the dominant and non-dominant foot may be important information when discussing lower limb phasic differences, but this was not shown in the present study.

## 6. Conclusions

Older adults had significantly increased coherence values in contralateral homologous muscle, unilateral heteronymous muscle, and contralateral heteronymous muscle–plantar flexor muscle pairs during static standing control. Furthermore, such coherence values were associated with increased COMacc, especially in the elderly. This co-modulation of intra- and interlimb muscle activity may contribute to the lack of standing stability characteristic of older adults. 

## Data Availability

The datasets generated during and/or analysed during the current study are available from the corresponding author.
